# *Pluchea indica* (L.) Less. Tea Ameliorates Hyperglycemia, Dyslipidemia, and Obesity in High Fat Diet-Fed Mice

**DOI:** 10.1155/2020/8746137

**Published:** 2020-06-03

**Authors:** Kittipot Sirichaiwetchakoon, Gordon Matthew Lowe, Sajeera Kupittayanant, Seekaow Churproong, Griangsak Eumkeb

**Affiliations:** ^1^School of Preclinic, Institute of Science, Suranaree University of Technology, 111 University Avenue, Suranaree Subdistrict, Muang District, Nakhon Ratchasima 30000, Thailand; ^2^School of Pharmacy and Biomolecular Sciences, Liverpool John Moores University, James Parsons Building, Byrom Street, Liverpool, UK; ^3^Institute of Medicine, Suranaree University of Technology, 111 University Avenue, Suranaree Subdistrict, Muang District, Nakhon Ratchasima 30000, Thailand

## Abstract

*Pluchea indica* (L.) Less. (*P. indica*) tea has been used for a health-promoting drink, especially in Southeast Asia. The effect of *P. indica* tea (PIT) on amelioration of hyperglycemia; dyslipidemia that was total cholesterol (TC), LDL-cholesterol (LDL-C), HDL-cholesterol (HDL-C), and triglyceride (TG); and obesity in high fat diet-induced (HFD) mice was investigated. Oral glucose tolerance test (OGTT) displayed that PIT at 400 and 600 mg/kg orally ameliorated hyperglycemia with a dose-dependent manner compared to the untreated group. Moreover, PIT at these dosages exhibited significantly lower TC, LDL-C, TG, and perigonadal fat weight in HFD treated mice compared to HFD mice (*P* < 0.05) with a dose-dependent manner. In contrast, HDL-C was higher than in the HFD group, but not a significant difference (*P* > 0.05). The PIT chemical analysis results demonstrated that PIT contained total phenolic content (TPC), total flavonoid content (TFC), 4-O-caffeoylquinic acid (4-CQ), 5-O-caffeoylquinic acid (5-CQ), 3,4-O-dicaffeoylquinic acid (3,4-CQ), 3,5-O-dicaffeoylquinic acid (3,5-CQ), 4,5-O-dicaffeoylquinic acid (4,5-CQ), beta-caryophyllene, and gamma-gurjunene that may play an important role in inhibiting hyperlipidemia and hyperglycemia. Also, histological analysis expressed that the mean area and amount of perigonadal fat adipocytes of PIT treated groups were significantly lower and higher than the HFD group (*P* < 0.05), respectively. The toxicity test of PIT at 600 mg/kg/day in mice showed that serum creatinine, alanine transaminase (ALT), alkaline phosphatase (ALP), and complete blood count (CBC) levels of HFD and PIT treated groups were not significantly different compared to the normal control diet group (NCD) (*P* > 0.05). These results suggest that PIT does not become toxic to the kidney, liver, and blood. In conclusion, PIT has the potential to develop into healthy food supplement or medicine for the prevention and treatment of hyperglycemic, hyperlipidemic, and obese patients.

## 1. Introduction

The incidence of diabetes mellitus, dyslipidemia, and obesity is currently increasing at a dramatic rate throughout the world. In 2015, an estimated 415 million people had type 2 diabetes worldwide, which is an equal rate compared among more than 90% of women and men [1]. Diabetes mellitus is a group of metabolic disorders characterized by hyperglycemia and insufficiency of secretion or action of endogenous insulin [2–4]. Diabetes is associated with long-term damage, dysfunction, and failure of different organs such as eyes, kidneys, nerves, heart, and blood vessels [5]. Diabetes ketoacidosis may occur in acute complications when blood sugar is higher, and it can become coma or death [6]. Moreover, diabetes mellitus is associated with dyslipidemia, which is one of the major risks for the provocation of cardiovascular disease (CVD) [7], and atherosclerosis which is a significant cause of morbidity and mortality worldwide [8]. Dyslipidemia is characterized by an abnormal amount of lipids, such as triglyceride, LDL-cholesterol, total cholesterol, and HDL-cholesterol in the blood [9]. Furthermore, diabetes and dyslipidemia are practically relevant to obesity [10–12]. Obesity is defined as abnormal or excessive fat accumulation caused by an imbalance of energy intake and expenditure. Obesity causes significant increase in the risk of morbidity and mortality [13]. Obesity can be treated by suppressing food intake, stimulating energy expenditure by regulating lipid metabolism, and reducing fat digestion and absorption [14].

Nowadays, current conventional medications for blood sugar and lipids modulating are widely used, but some drugs have limited efficacies and critical adverse effects, such as rhabdomyolysis from HMG-CoA reductase inhibitors [15] and hypoglycemia from insulin and sulfonylurea [16, 17]. Therefore, the use of herbal supplements or alternative medicines, which may have minor adverse effects and are often cheaper and easily consumable [18], has become increasingly popular during the last decade [19].

*P. indica* (Asteraceae) leaves have been used for a health-promoting drink [20] and in folk medicine, especially in Southeast Asia, including Thailand. The plant has various biological activities, such as a diuretic, and antihyperglycemic activities of its methanolic extract [21, 22]. Moreover, PIT also had antiadipogenesis in 3T3-L1 cell and lipase enzyme inhibition effects [23]. Therefore, PIT has the potential to develop to be a health-promoting drink and herbal medicine for hyperglycemia and dyslipidemia prevention.

This study aimed to investigate the effect of PIT on hyperglycemia and dyslipidemia prevention in mice. Besides, the antiobesity and toxicity of this tea in mice were also studied.

## 2. Materials and Methods

### 2.1. Plant Materials

PIT was supported and prepared by The Crystal Biotechnology Co., Thailand, and Suranaree University of Technology. In brief, fresh herbs of *P. indica* were collected from Nakhon Ratchasima and northeast region of Thailand. The plant specimen was authenticated by Dr. Paul J Grote, and the identification was made in comparison with the voucher specimen (BKF 194428) and deposited at Forest Herbarium, National Park, Wildlife, and Plant Conservation Department, Ministry of Natural Resources and Environment, Thailand. These plants were washed thoroughly; then they were ground and dried in an oven at 80°C for 120 min. Tea was freshly prepared daily by brewing 3g dried extract in 80°C distilled water 100 mL for 5 min and was filtered by Whatman No. 1 filter paper to complete 3%°(w/v) PIT extract.

### 2.2. Chemicals and Reagents

A total of 10% neutral buffered formalin solution, sucrose, D-(+)-glucose, xylenes, Mayer's hematoxylin solution, eosin Y solution, simvastatin, and glibenclamide were obtained from Sigma-Aldrich Chemical Co. (St. Louis, MO, USA) and 60% fat calories mouse diet or high fat diet (% composition: protein 20%, fat 36%, fiber 0%, ash 3.5%, moisture < 10%, and carbohydrate 35.7%) was purchased from Bio-Serv (Frenchtown, NJ, USA). 5-O-Caffeoylquinic acid (5-CQ), 4,5-O-dicaffeoylquinic acid (4,5-CQ), 4-O-caffeoylquinic acid (4-CQ), 3,5-O-dicaffeoylquinic acid (3,5-CQ), and 3,4-O-dicaffeoylquinic acid (3,4-CQ) were purchased from Chengdu Biopurify Phytochemicals Ltd., (Sichuan, China). All reagents used were analytical grade.

### 2.3. Total Phenolic Content (TPC), Total Flavonoid Content (TFC), Liquid Chromatography-Mass Spectrometry/Mass Spectrometry (LC-MS/MS) Analysis, and Gas Chromatography-Mass Spectrometry (GC-MS) Analysis

The active chemical compounds in PIT were analyzed by TPC, TFC, LC-MS/MS, and GC-MS. Total phenolic content was measured by the Folin–Ciocalteu assay following Singleton and Rupasinghe et al.'s method [24]. Similarly, an aluminium chloride colorimetric assay was performed to determine total flavonoid content [25]. Furthermore, the major chemical compounds of the PIT were analyzed using the LC-MS/MS instrument, as previously reported by Sirichaiwetchakoon et al. [23]. In brief, The LC-MS/MS system was combined from Agilent HPLC 1290 Infinity and mass analyzer 6490 Triple Quad LC/MS Agilent Technologies, with electrospray ionization (ESI) source system, consisting of an autosampler, a binary pump, and vacuum degasser. Agilent ZORBAX Rapid Resolution High Definition (RRHD) SB-C18, 2.1 mm id × 150 mm (1.8 *µ*m), was used for chromatographic separation and mobile phase system used solvents A and B, which consisted of 1% formic acid in water and in acetonitrile, respectively. A combination of both solvents in LC system was set at a ratio of solvent A : solvent B, 100 : 0 with gradient elution—from 30% solvent B at 10 min and 100% solvent B at 30 min at a flow-rate of 0.2 mL·min^−1^. The sample injection volume was set at 5 *µ*L, and the temperature was maintained at 25°C. The solutions of 4-CQ, 5-CQ, 3,4-CQ, 3,5-CQ, and 4,5-CQ were used as standards. Moreover, the GC-MS analysis was performed by using Bruker Gas Chromatography Model 450GC equipped with Bruker 320MS. Analysts were separated on a column: Rtx-5MS capillary column (30 m × 0.25 mm, fused silica 0.25 *µ*m). The following temperature program was set up at 50–250°C with 2 increasing steps. Oven initial temperature was 50°C for 1 min, then increased to 120°C at a rate of 5°C/min, and held at the 120°C for 40 min. In the final step, the column temperature was increased up to 250°C at a rate of 3°C/min. The total running time was 98.33 min, and the carrier gas was helium (1 mL·min^−1^). Injector volume was 1 *μ*l, and injector temperatures were held at 250°C. Compound identification was done by comparing it with NIST Mass Spectral Library.

### 2.4. Animals and Experimental Design

Ninety adult male mice (ICR mouse), aged about 8 weeks and weighing 30–40 g, were used in these experiments. These mice were divided into 2 groups. The first group was 40 mice for an oral glucose tolerance test. The second group was 50 mice for lipid profile and toxicological examination. All mice were obtained from the Animal Care Building, Suranaree University of Technology, Nakhon Ratchasima, Thailand. The experimental protocol was approved in accordance with a guideline for the care and use of laboratory animals by the animal care and use committee (ACUC), Suranaree University of Technology. The approval number of institutional authorities on the care and use of animals was 7/2560. Mice were housed in a light, humidity, and temperature controlled room (light on 12 h/day, temperature 25 ± 0.5°C, and the moisture 40% ± 2%) at the animal care building at Suranaree University of Technology, Nakhon Ratchasima, Thailand. The mice had free access to food pellets and water except when fasted before blood collection and necropsy.

### 2.5. An Oral Glucose Tolerance Test (OGTT)

An oral glucose tolerance test was performed to measure how well the body can process a more considerable amount of sugar. This test was carried out using the method described by Clemmensen et al. with slight modifications [26]. After acclimation for 2 weeks, mice were randomly divided into 4 groups (*n* = 10). Then, fasting for 16 h, blood from all mice was collected for measuring blood glucose before each group received single oral administration of D-glucose at 2 g/kg body weight plus the following test agent. The control group (CON) was fed water. The positive control group was fed glibenclamide at 10 mg/kg/day (GLI). The *P. indica* tea at a low dose (PIL) and high dose (PIH) treated groups were fed PIT at 400 and 600 mg/kg/day, respectively. Blood glucose was measured by an Accu-Chek glucometer (Roche, Basel, Switzerland) using Accu-Chek test strips at 0, 15, 30, 60, and 120 min that initiated from the oral glucose administration.

### 2.6. The Lipid Profile and Toxicological Testing

**Figure 1:**
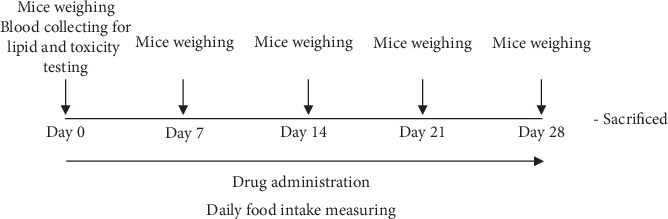
Schematic drawing of the experimental protocol. (a) Lipid and toxicity analysis. (b) Relative organ analysis. (c) Perigonadal fat analysis.

The effect of PIT on serum lipid profile and toxicity experiments were performed as previously described by Vaghasiya et al. and Kuo et al. with little modifications [27, 28]. The flow diagram of the experimental design is shown in Figure 1. Shortly, mice were randomly divided into 5 groups (*n* = 10). The normal control diet group (NCD) was fed a normal mouse diet and water. The high fat diet group (HFD) was fed a high fat diet and water. The positive control group, simvastatin (SIM), was fed a high fat diet and simvastatin at 20 mg/kg/day. The PIT at a low dose (PIL) and high dose (PIH) treated groups were fed high fat diet and PIT at 400 and 600 mg/kg/day, respectively. The experimentation was performed for 4 weeks. At the end of the treatment period, all mice were sacrificed under thiopental sodium anesthesia and subjected to necropsy. The blood was collected to analyze total cholesterol (TC), LDL-cholesterol (LDL-C), triglyceride (TG), and HDL-cholesterol (HDL-C). Besides, creatinine, alanine transaminase (ALT), alkaline phosphatase (ALP), and complete blood count (CBC) were measured for toxicity testing.

### 2.7. Measurement of Body Weight, Food Intake, Relative Organ, and Perigonadal Fat Weight

The body weight, food intake, relative organ, and perigonadal fat weight were measured using Han et al.'s method [29]. In brief, the body weight of all mice was measured every week. Food intake was assessed daily, and the average daily food intake was calculated. At the end of the experiment, mice were sacrificed. Then, the liver, heart, kidney, lung, spleen, and perigonadal fat were collected, and weights were measured. The relative organ and perigonadal fat weight per 100 g of total body weight of each mouse were calculated as follows:

(1) $$\begin{array}{c} \text{relative organ weight}\left(\text{g}/100\text{ g body weight}\right)=\frac{\text{weight of mouse organ}\left(\text{g}\right)\times 100}{\text{mouse body weight}\left(\text{g}\right)}. \end{array}$$

### 2.8. Histological Analysis

The perigonadal fat pad histological analysis was performed as previously described by Kim et al. with little modifications [30]. In brief, after treatment for 4 weeks, all mice were sacrificed. Next, the perigonadal fat pad was collected and preserved in 10% (w/v) neutral phosphate buffer formaldehyde. The tissues were fixed in 10% neutral buffered formalin, embedded in paraffin, and cut with a microtome at 5 *µ*m. The sectioned tissues were placed in xylenes and rehydrated through serial alcohol gradients (100%, 95%, 90%, 80%, 70%, and 50%, 2 min each). Hematoxylin and eosin were used for staining. The histopathology of the tissue slide was examined under a light microscope. The number of adipocytes per each field and the central area of each adipocyte were counted and analyzed.

### 2.9. Statistical Analysis

All data were presented as the mean ± SEM. The statistically significant differences between groups of food intake, mouse weight, blood toxicity testing, epididymal fat pads, and relative organ weight were analyzed by ANOVA with a Tukey's HSD post hoc test. Paired Student's *t*-test was used to compare the differences in serum lipid profile (TC, LDL, HDL, and TG) analysis between pre- and posttreatment groups. Then, a significant difference between each group was compared using ANCOVA. Tukey's HSD post hoc test at *P* < 0.05, which means sharing the different superscript letters, was also considered a statistically significant difference between each group [31, 32].

## 3. Results

### 3.1. Total Phenolic Content (TPC), Total Flavonoid Content (TFC), LC-MS/MS Analysis, and GC-MS Analysis

**Figure 2:**
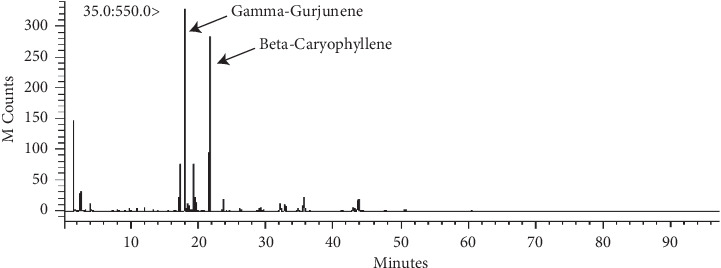
GC-MS chromatogram of compounds in PIT.

The TPC results indicated that the PIT contained 107.95 ± 4.87 mg GAE/g of dry weight of total phenolic content. Moreover, TFC analyzed demonstrated that PIT was comprised of 95.33 ± 0.48 mg CE/g of the dry weight of total flavonoid content. The MRM transition chromatograms at *m/z* 353 ⟶ 191.0 displayed that 4-CQ and 5-CQ were detected in the PIT extract. Furthermore, 3,4-CQ, 3,5-CQ, and 4,5-CQ were discovered at *m/z* 515 ⟶ 353 (Table 1). These results are virtually the same quantity as the previous result of Sirichaiwetchakoon et al. [23]. The highest of these compounds was 3,5-CQ and it was found that a PIT concentration of 1500 *µ*g·mL^−1^ consisted of 3,5-CQ at 169.93 *µ*g·mL^−1^. The GC-MS analysis of the PIT is presented in Figure 2 and Table 2. The results showed that the PIT contained 31 compounds, and the abundant primary compounds were beta-caryophyllene and gamma-gurjunene with %area at 33.56% and 25.90%, respectively.

### 3.2. Oral Glucose Tolerance Test (OGTT)

**Figure 3:**
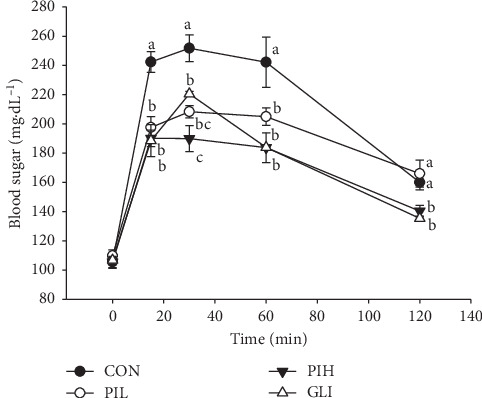
Effect of PIT on an oral glucose tolerance test. CON = control; PIL = PIT at 400 mg/kg; PIH = PIT at 600 mg/kg; GLI = glibenclamide at 10 mg/kg. Data are expressed as means ± SEM (*n* = 10). Means with the different superscript letters are significantly different from each other (Tukey's HSD test, *P* < 0.05).

Antihyperglycemic effects of PIT were evaluated in normal mice. Dosages of PIT at 400 and 600 mg/kg were compared with glibenclamide 10 mg/kg, which was used as a positive control. The blood glucose level was measured at 0, 15, 30, 60, and 120 min, respectively.  Figure 3 showed that the blood glucose level of PIL, PIH, and GLI groups was significantly lower than the untreated group from 15 to 60 min (*P* < 0.05). At 30 min, the high dose of PIT (PIH) expressed a significantly lower blood glucose level compared to GLI (*P* < 0.05). Besides, the blood sugar levels of PIH and GLI groups were considerably lower than those of CON and PIL at 120 min (*P* < 0.05).

### 3.3. Effect of PIT on Serum Lipid Profiles

**Figure 4:**
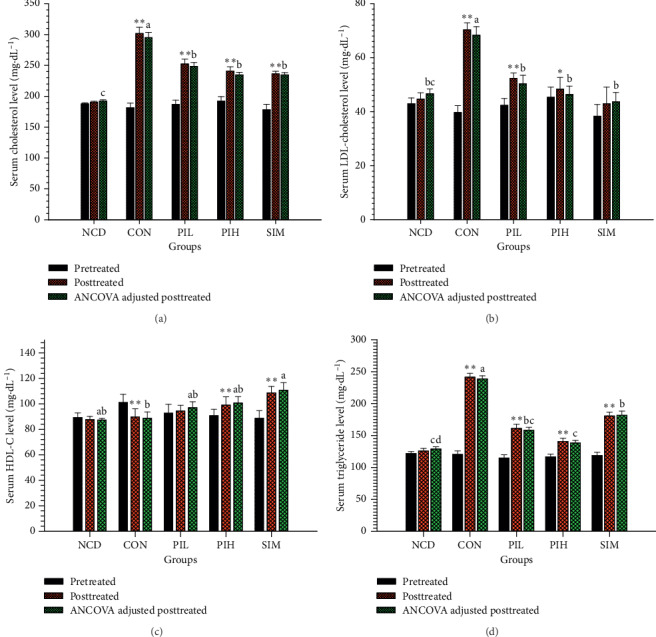
Effect of PIT on serum lipid profile. NCD = normal control diet group; HFD = high fat diet group; PIL = PIT at 400 mg/kg/d; PIH = PIT at 600 mg/kg/d; SIM = simvastatin at 20 mg/kg/d. (a) Serum cholesterol, (b) serum LDL-C, (c) serum TG, and (d) serum HDL-C. Data are expressed as means ± SEM (*n* = 10). The significant differences between pre- and posttest in each group were compared using paired Student's *t*-test at ^*∗*^*P* < 0.05 and ^*∗∗*^*P* < 0.01. A significant difference between ANCOVA adjusted the posttreated level in each group, which means sharing the different superscript letters, and was compared using ANCOVA and Tukey's HSD post hoc test at *P* < 0.05.

The effect of PIT on serum lipid profile had been revealed. The TC, LDL-C, and TG levels of the HFD group were significantly higher than the NCD group (*P* < 0.05; Figures 4(a), 4(b), and 4(d)). The serum TC levels in the PIL, PIH, and SIM groups were 257.40 ± 21.39 mg·dL^−1^, 242.50 ± 12.82 mg·dL^−1^, and 237.50 ± 12.05 mg·dL^−1^, respectively, significantly lower than the HFD which was 306.80 ± 27.37 mg·dL^−1^ at week 4 (*P* < 0.05; Figure 4(a)). Furthermore, Figure 4(b) showed the serum LDL-C levels of PIL (53.16 ± 10.62 mg·dL^−1^), PIH (47.43 ± 4.17 mg·dL^−1^), and SIM (42.48 ± 2.54 mg·dL^−1^) were significantly lower than the HFD (73.12 ± 6.83 mg·dL^−1^) at week 4 (*P* < 0.05). Interestingly, the serum TG level of the PIH treated group (147.25 ± 9.13 mg·dL^−1^) was significantly lower than the positive control (SIM, 180.75 ± 19.30 mg·dL^−1^) and HFD groups (240.60 ± 27.02 mg·dL^−1^) (*P* < 0.05), while there was no significant difference from the PIL group (157.45 ± 10.15 mg·dL^−1^) (Figure 4(d)). The result of the serum HDL-C level exhibited that the HDL-C levels at week 4 of PIL and PIH groups were higher than the HFD group, but it was not a significant difference (*P* > 0.05, Figure 4(c)). Also, the HDL-C level of the SIM group was significantly higher than the HFD group (*P* < 0.05).

### 3.4. Effect of PIT on Food Intake and Body Weight

**Figure 5:**
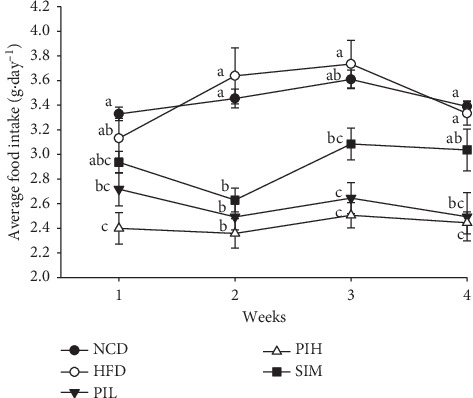
Effect of PIT on average food intake. NCD = normal control diet group; HFD = high fat diet group; PIL = PIT at 400 mg/kg/d; PIH = PIT at 600 mg/kg/d; SIM = simvastatin at 20 mg/kg/d. Data are expressed as means ± SEM (*n* = 10). Means with the different superscript letters in the same week are significantly different from each other (Tukey's HSD test, *P* < 0.05).

The average food intake of mice was investigated.  Figure 5 shows the effect of PIL and PIH on food intake compared to NCD, HFD, and SIM groups. PIL and PIH showed significantly lower food intake than the HFD group from the first week until the end of the experiment (*P* < 0.05).

**Figure 6:**
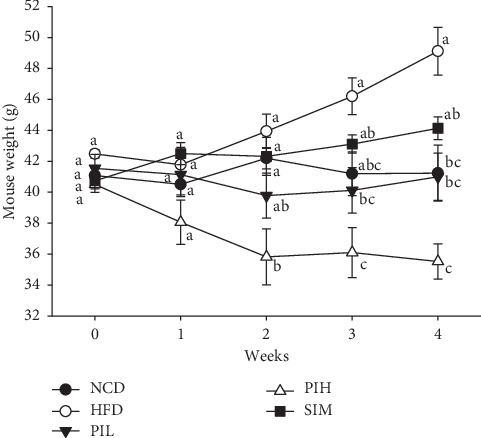
Effect of PIT on mouse weight. NCD = Normal control diet group; HFD = High fat diet group; PIL = PIT at 400 mg/kg/d; PIH = PIT at 600 mg/kg/d; SIM = simvastatin at 20 mg/kg/d. Data are expressed as means ± SEM (*n* = 10). Means with the different superscript letters in the same week are significantly different from each other (Tukey's HSD test, *P* < 0.05).

The body weight of mice in all groups was measured every week. The result was consistent with the average food intake result (Figure 6). The mouse's weight of the HFD group was significantly heavier than the NCD group at week 4 (*P* < 0.05, Figure 6). Apart from this, the HFD group exhibited TC, TG, and DL-C significantly higher than the NCD group (*P* < 0.05, Figure 4). For this reason, these findings provide evidence that these HFD mice are obese. At week 2, PIH showed significantly lower body weight than the HFD, NCD, and SIM group (*P* < 0.05), but not a significant difference from PIL (*P* > 0.05). Moreover, the body weight of the PIL group was significantly lower than the HFD group from weeks 3 to 4 (*P* < 0.05).

### 3.5. Effect of PIT on Relative Organ Weight

The relative organ weights of liver, heart, kidney, lung, and spleen of mice after feeding with HFD and HFD plus PIL or PIH or SIM are shown in Table 3. The relative weights of the liver, heart, kidney, lung, and spleen of PIL, PIH, and SIM groups were not significantly different from the NCD and HFD groups (*P* > 0.05).

### 3.6. Effect of PIT on Biochemical Parameters in Mice Serum

**Figure 7:**
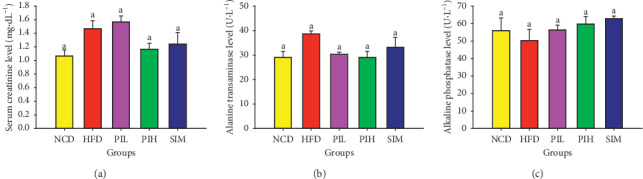
Effect of PIT on biochemical parameters in serum. NCD = normal control diet group; HFD = high fat diet group; PIL = PIT at 400 mg/kg/d; PIH = PIT at 600 mg/kg/d; SIM = simvastatin at 20 mg/kg/d. (a) Serum creatinine, (b) serum ALT, and (c) serum ALP. Data are expressed as means ± SEM (*n* = 10). Means with the different superscript letters are significantly different from each other (Tukey's HSD test, *P* < 0.05).

**Figure 8:**
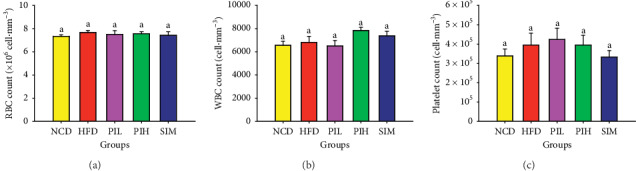
Effect of PIT on complete blood count. NCD = normal control diet group; HFD = high fat diet group; PIL = PIT at 400 mg/kg/d; PIH = PIT at 600 mg/kg/d; SIM = simvastatin at 20 mg/kg/d. (a) Red blood cell count, (b) white blood cell count, and (c) platelet count. Data are expressed as means ± SEM (*n* = 10). Means with the different superscript letters are significantly different from each other (Tukey's HSD test, *P* < 0.05).

The serum creatinine, ALT, ALP, and the CBC were measured to investigate the toxicity of PIT on the kidney, liver, and blood.  Figure 7(a) showed that the serum creatinine levels of PIL, PIH, and SIM groups were not significantly different compared to NCD and HFD groups (*P* > 0.05). These results suggest that PIL, PIH, and SIM may not be toxic to the kidney. Besides, the serum ALT and ALP of the PIL, PIH, and SIM groups were not significantly different from NCD and HFD groups (*P* > 0.05, Figures 7(b) and 7(c)). These results imply that PIL, PIH, and SIM should not be toxic to the liver. The CBC testing provides essential information regarding three major types of cells in the blood: RBC count, WBC count, and platelets. Figures 8(a)–8(c) display RBC count, WBC count, and platelets, respectively. These results showed that the CBC of all treated groups was not significantly different compared to NCD and HFD groups (*P* > 0.05).

### 3.7. Effect of PIT on Perigonadal Fat

**Figure 9:**
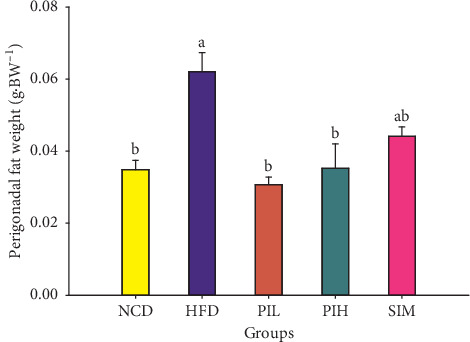
Effect of PIT on perigonadal fat weight. NCD = normal control diet group; HFD = high fat diet group; PIL = PIT at 400 mg/kg/d; PIH = PIT at 600 mg/kg/d; SIM = simvastatin at 20 mg/kg/d. Data are expressed as means ± SEM (*n* = 10). Means with the different superscript letters are significantly different from each other (Tukey's HSD test, *P* < 0.05).

**Figure 10:**
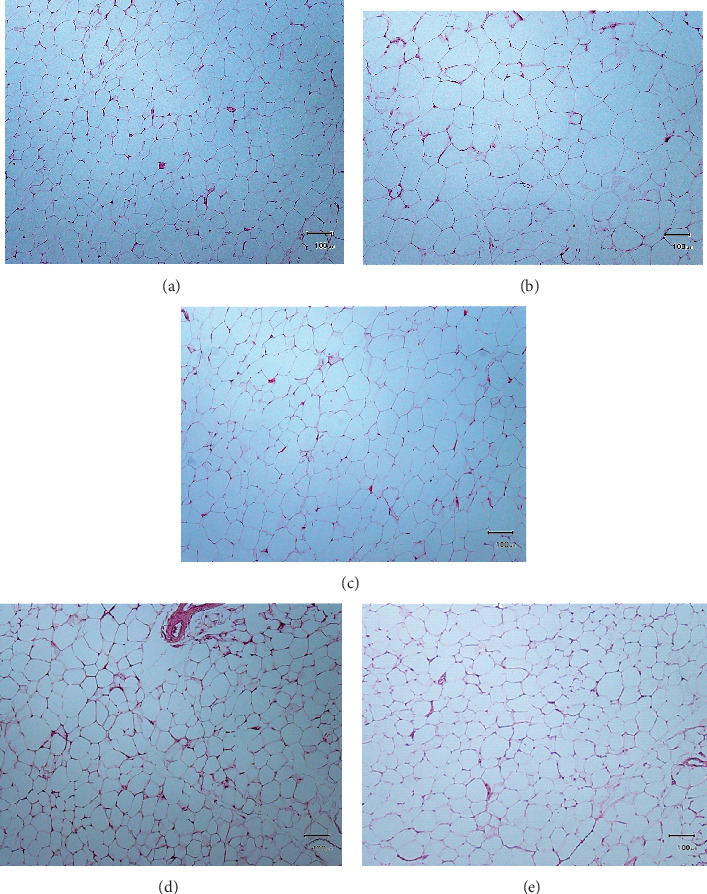
Microscopic imaging of perigonadal fat tissue after Hematoxylin & Eosin staining of samples. (a) Normal control diet group; (b) high fat diet group; (c) PIT at 400 mg/kg/d; (d) PIT at 600 mg/kg/d; (e) simvastatin at 20 mg/kg/d. Original magnification × 10 (scale bars = 100 *µ*m).

**Figure 11:**
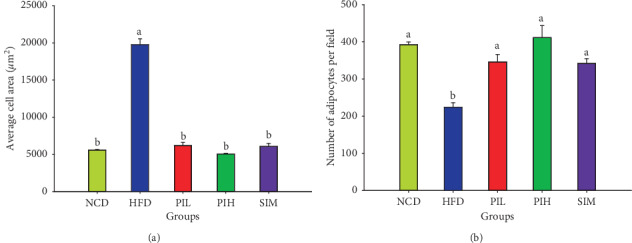
Effect of PIT on perigonadal fat tissues. NCD = normal control diet group; HFD = high fat diet group; PIL = PIT at 400 mg/kg/d; PIH = PIT at 600 mg/kg/d; SIM = simvastatin at 20 mg/kg/d. (a) Adipocytes size (area) and (b) number of adipocytes per field. Data are expressed as means ± SEM (*n* = 10). Means with the different superscript letters are significantly different from each other (Tukey's HSD test, *P* < 0.05).

The perigonadal fat weight result is demonstrated in Figure 9. The perigonadal fat weights of PIL and PIH groups were significantly lower than the HFD group (*P* < 0.05), but not a significant difference from NCD and SIM groups (*P* > 0.05). The H&E staining of perigonadal fat photographs is shown in Figure 10. The adipocytes size (area) and the number of adipocytes per field are demonstrated in Figures 11(a) and 11(b), respectively. The mean area and the number per field of adipocytes of PIL and PIH groups were significantly lower and higher than the HFD group (*P* < 0.05), respectively. However, these parameters were not significant differences compared to NCD and SIM groups (*P* > 0.05).

## 4. Discussion

Obesity is occurring by an imbalance between energy consumption and expenditure. Obesity is a major risk factor of various diseases, especially type 2 diabetes, dyslipidemia, and cardiovascular disease [33, 34].

High fat diet feeding in mice has been widely used as a model for dyslipidemia studies of antidyslipidemia evaluation because it can induce obesity and dyslipidemia. PIT has been used for a health-promoting drink [20] that has the potential to protect hyperglycemia, obesity, and dyslipidemia.

The results from this study showed antihyperglycemic effect of PIT using an oral glucose tolerance test compared with conventional medicine glibenclamide. The results showed that PIT at 400 and 600 mg/kg showed a significant hypoglycemic effect in mice after feeding with glucose. Interestingly, PIH reduced the plasma glucose level to 190 ± 8.84 mg·dL^−1^, which is significantly lower than the standard drug, glibenclamide, at 30 min. These results are in substantial agreement with Pramanik et al. [21] where the methanolic extract of *P. indica* leaves used in normal and streptozotocin-induced diabetic rats can show hypoglycemic effect. The PIT chemical compound from LC-MS/MS results demonstrated that PIT contained 4-CQ, 5-CQ, 3,4-CQ, 3,5-CQ, and 4,5-CQ. These results are in substantial agreement with those of previous reports in that one of the active ingredients in *P. indica* (L.) is caffeoylquinic acid derivatives [20, 23, 35]. These derivatives could inhibit intestinal maltase, which might have delayed postprandial hyperglycemia [35, 36]. Likewise, it had been reported that the caffeoylquinic acid derivatives, which are contained in this plant, might have benefits for the treatment of diabetes by alleviating hyperglycemia. This effect is caused by an increase in insulin sensitivity via activation of AMPK–AS160–GLUT4 pathway in skeletal muscles and alpha-glucosidase inhibition, which inhibits the formation of advanced glycation end products (AGEs), and gluconeogenesis in the liver [37–41]. Apart from this, the other main components of PIT from GC-MS analysis were beta-caryophyllene, which had been reported about antihyperglycemia through enhancing insulin release in diabetic rats [42, 43], and gamma-gurjunene. These findings lead us to believe that caffeoylquinic acid derivatives and beta-caryophyllene from PIT may play an important role in inhibiting hyperglycemia by intestinal maltase inhibition, increasing insulin release and sensitivity, or inhibiting alpha-glucosidase which results in AGEs and gluconeogenesis inhibition.

Moreover, HFD-fed mice treated with PIT (400 and 600 mg/kg daily) had significantly lower TG, TC, and LDL-C than the HFD group, whereas HDL-C was higher than HFD group, but not significantly different. The effect of PIH and PIL on lowering mouse weight and lipid profile corresponded with food intake results in that these extracts could reduce food intake. Furthermore, the extracts could decrease perigonadal fat pad weight and adipocyte size and increase the number of adipocytes per field of testing mice. These results are in substantial agreement with our previous findings that PIT inhibits lipids and carbohydrate accumulation in adipocytes and interrupts pancreatic lipase activity [23] that can lead to a decrease in serum lipid profile and obesity. Moreover, the previous study found that caffeoylquinic acid derivatives, which appeared in PIT [20, 23, 35], could suppress diet-induced body fat accumulation by downregulating *SREBP-1c* and related molecules in C57BL/6J mice [44]. Nugroho et al. [45] reported that oral administration of caffeoylquinic acid-rich *L. stenocephala* in BuOH fraction form in rat could decrease the rat body weight to the level of the untreated group and reduce abdominal fat pad weight. Moreover, the caffeoylquinic acid had been reported to have antihyperlipidemic mechanism by increasing the expression of PPARalpha and PPARdelta, suppressing adiponectin, and upregulation of LPL and AMPK activities [46–48]. Furthermore, it had been reported that beta-caryophyllene, one of the main components in the PIT, could attenuate palmitate-induced lipid accumulation through AMPK signaling and prevent atherosclerosis in hypercholesterolemic rats [49–52]. These pieces of evidence lead us to believe that PIT may have an antidyslipidemia effect because of increasing the expression of PPARalpha and PPARdelta, suppressing adiponectin, and the upregulation of LPL and AMPK activities.

The toxicity results of PIT on relative organ weight and biochemical parameters revealed that the relative weights of liver, heart, kidney, lung, and spleen and ALP, ALT, creatinine, RBC, WBC, and platelet of PIT treated groups were not significant differences from the NCD and HFD groups (*P* > 0.05). These results are in substantial agreement with those of Pramanik et al. [21] that a methanolic extract of *P. indica* in the rat was safe to use even at the doses of 3200 mg/kg of body weight orally. Noticeably, the dosages of PIT in the experiment can be converted to a human dose by a simple, practical guide for dosage conversion between animals and humans [53]. From the formula, PIT at 400 and 600 mg/kg/d in mice are approximately 35.52 and 48.78 mg/kg/d in humans, respectively. These dosages lead us to believe that PIT would be safe to a human for drinking once daily for 4 weeks.

## 5. Conclusions

These findings provide evidence that PIT would ameliorate hyperglycemia and dyslipidemia and reduce weight gain of high fat diet mice. Furthermore, the results also exhibited the blood and vital organs' safety of the PIT. So, PIT might have the potential to be developed as a healthy food supplement for antihyperglycemia, antidyslipidemia, and antiobesity. However, further investigation of the pharmacological activity of these active ingredients and safety in humans is required.

## Figures and Tables

**Table 1:** A main chemical constituent of PIT that was analyzed by LC-MS/MS analysis.

Main chemical constituent	Detection
4-O-Caffeoylquinic acid (4-CQ)	+
5-O-Caffeoylquinic acid (5-CQ)	+
3,4-O-Dicaffeoylquinic acid (3,4-CQ)	+
3,5-O-Dicaffeoylquinic acid (3,5-CQ)	+
4,5-O-Dicaffeoylquinic acid (4,5-CQ)	+

+ means those compounds have been detected.

**Table 2:** Compounds in PIT that was analyzed by GC-MS analysis.

Compounds	RT (min)	Area (%)
Methane, thiobis-	1.555	4.89
2-Propenoic acid	2.600	4.05
Hexanal	3.402	0.09
Dimethyl sulfoxide	4.005	1.14
2,4-Heptadienal, (E,E)-	8.111	0.28
3,5-Octadien-2-one, (E,E)-	9.766	0.32
2-Heptanone, 6-methyl-	10.825	0.21
2,5-Furandione, 3-(1,1-dimethylethyl)-	12.032	0.26
Decane	13.354	0.09
Beta-cyclocitral	14.018	0.07
2H-2,4a-Ethanonaphthalene, 1,3,4,5,6,7-hexahydro-	17.240	4.10
Gamma-gurjunene	18.033	25.90
Alpha-cubebene	18.183	0.18
Aromadendrene, dehydro-	18.517	0.71
(+)-Cyclosativene	18.985	0.33
Copaene	19.378	5.14
Alpha-patchoulene	19.629	1.04
Beta-cubebene	20.035	0.10
Dodecane	20.495	0.09
Beta-caryophyllene	21.755	33.56
Beta-selinene	23.706	1.86
Beta-ionone	26.190	0.61
Drimenol	29.204	0.74
Delta-cadinene	29.464	0.26
2(4H)-Benzofuranone, 5,6,7,7a-tetrahydro-4,4,7a-trimethyl-	29.687	0.54
Caryophyllene oxide	32.180	2.08
1,5,5,8-Tetramethyl-12-oxabicyclo[9.1.0]dodeca-3,7-diene	32.858	1.78
4,11,11-Trimethyl-8-methylenebicyclo[7.2.0]undec-4-ene	34.828	0.75
Caryophyllene oxide	35.706	4.04
Tetracyclo[6.3.2.0(2,5).0(1,8)]tridecan-9-ol,4,4-dimethyl-	43.841	4.74
Isopropyl palmitate	78.750	0.05

**Table 3:** Effect of PIT on relative organ weight.

Groups	Relative weight (g/100 g body weight)				
	Liver	Heart	Kidney	Lung	Spleen
NCD	3.66 ± 0.27^a^	0.43 ± 0.04^a^	1.64 ± 0.03^a^	0.57 ± 0.06^a^	0.31 ± 0.02^a^
HFD	3.80 ± 0.18^a^	0.45 ± 0.01^a^	1.43 ± 0.13^a^	0.51 ± 0.02^a^	0.27 ± 0.07^a^
PIL	3.96 ± 0.13^a^	0.48 ± 0.02^a^	1.67 ± 0.14^a^	0.57 ± 0.09^a^	0.30 ± 0.03^a^
PIH	3.93 ± 0.04^a^	0.46 ± 0.01^a^	1.72 ± 0.12^a^	0.63 ± 0.04^a^	0.33 ± 0.05^a^
SIM	3.63 ± 0.16^a^	0.44 ± 0.02^a^	1.44 ± 0.05^a^	0.54 ± 0.02^a^	0.28 ± 0.02^a^

NCD = normal control diet group; HFD = high fat diet group; PIL = PIT at 400 mg/kg/d; PIH = PIT at 600 mg/kg/d; SIM = simvastatin at 20 mg/kg/d. Data are expressed as means ± SEM (*n* = 10). Means with the same superscript alphabet in the same column are not significantly different from each other (Tukey's HSD test, *P* < 0.05).

## Data Availability

The datasets used and analyzed during the current study are available from the corresponding author on reasonable request.
